# Supplementary material for: Iterative improvement in the automatic modular design of robot swarms
Source: PeerJ Comput Sci. 2020 Dec 7;6:e322. doi: 10.7717/peerj-cs.322 (PMC7924708; doi:10.7717/peerj-cs.322)
Supplement: Supplemental Information 3 [file peerj-cs-06-322-s003.zip › argos3/doc/api/standalone/a00531_source.html]

ARGoS: plugins/robots/generic/simulator/leds\_default\_actuator.h Source File


- Main Page
- Related Pages
- Namespaces
- Classes
- Files

- File List
- File Members

# plugins/robots/generic/simulator/leds\_default\_actuator.h

Go to the documentation of this file.

```
00001 
00007 #ifndef LEDS_DEFAULT_ACTUATOR_H
00008 #define LEDS_DEFAULT_ACTUATOR_H
00009 
00010 #include <string>
00011 #include <map>
00012 
00013 namespace argos {
00014    class CLEDsDefaultActuator;
00015    class CLEDMedium;
00016 }
00017 
00018 #include <argos3/plugins/robots/generic/control_interface/ci_leds_actuator.h>
00019 #include <argos3/plugins/simulator/entities/led_equipped_entity.h>
00020 #include <argos3/core/simulator/actuator.h>
00021 
00022 namespace argos {
00023 
00024    class CLEDsDefaultActuator : public CSimulatedActuator,
00025                                 public CCI_LEDsActuator {
00026 
00027    public:
00028 
00029       CLEDsDefaultActuator();
00030 
00031       virtual ~CLEDsDefaultActuator() {}
00032 
00033       virtual void SetRobot(CComposableEntity& c_entity);
00034 
00035       virtual void Init(TConfigurationNode& t_tree);
00036       virtual void Update();
00037       virtual void Reset();
00038       virtual void Destroy();
00039 
00040    private:
00041 
00042       CLEDEquippedEntity* m_pcLEDEquippedEntity;
00043       CLEDMedium*         m_pcLEDMedium;
00044 
00045    };
00046 
00047 }
00048 
00049 #endif
```

---

Generated on 10 Jul 2018 for ARGoS by 
 1.6.1
